# Insects in confined swine operations carry a large antibiotic resistant and potentially virulent enterococcal community

**DOI:** 10.1186/1471-2180-11-23

**Published:** 2011-01-26

**Authors:** Aqeel Ahmad, Anuradha Ghosh, Coby Schal, Ludek Zurek

**Affiliations:** 1 Department of Entomology, Kansas State University, Manhattan, KS, USA; 2 Department of Diagnostic Medicine and Pathobiology, Kansas State University, Manhattan, KS, USA; 3 Department of Entomology, North Carolina State University, Raleigh, NC, USA; 4 Monsanto Company, St. Louis, MO 63167, USA

## Abstract

**Background:**

Extensive use of antibiotics as growth promoters in the livestock industry constitutes strong selection pressure for evolution and selection of antibiotic resistant bacterial strains. Unfortunately, the microbial ecology and spread of these bacteria in the agricultural, urban, and suburban environments are poorly understood. Insects such as house flies (*Musca domestica*) and German cockroaches (*Blattella germanica*) can move freely between animal waste and food and may play a significant role in the dissemination of antibiotic resistant bacteria within and between animal production farms and from farms to residential settings.

**Results:**

Enterococci from the digestive tract of house flies (*n *= 162), and feces of German cockroaches (*n *= 83) and pigs (*n *= 119), collected from two commercial swine farms were isolated, quantified, identified, and screened for antibiotic resistance and virulence. The majority of samples (93.7%) were positive for enterococci with concentrations 4.2 ± 0.7 × 10^4 ^CFU/house fly, 5.5 ± 1.1 × 10^6 ^CFU/g of cockroach feces, and 3.2 ± 0.8 × 10^5 ^CFU/g of pig feces. Among all the identified isolates (n = 639) *Enterococcus faecalis *was the most common (55.5%), followed by *E. hirae *(24.9%), *E. faecium *(12.8%), and *E. casseliflavus *(6.7%). *E. faecalis *was most prevalent in house flies and cockroaches, and *E. hirae *was most common in pig feces. Our data showed that multi-drug (mainly tetracycline and erythromycin) resistant enterococci were common from all three sources and frequently carried antibiotic resistance genes including *tet*(M) and *erm*(B) and Tn*916*/*1545 *transposon family. *E. faecalis *frequently harbored virulence factors *gelE, esp, *and *asa1*. PFGE analysis of selected *E. faecalis *and *E. faecium *isolates demonstrated that cockroaches and house flies shared some of the same enterococcal clones that were detected in the swine manure indicating that insects acquired enterococci from swine manure.

**Conclusions:**

This study shows that house flies and German cockroaches in the confined swine production environment likely serve as vectors and/or reservoirs of antibiotic resistant and potentially virulent enterococci and consequently may play an important role in animal and public health.

## Background

Antibiotic resistance is a serious public-health problem; reduced effectiveness of antibiotics results in greater patient mortality rates, prolonged hospitalization and increased healthcare costs. The economic impact of antibiotic resistance has been estimated between $5 and $24 billion annually in the United States alone [1]. Extensive use of antibiotics, especially as growth promoters, in the animal industry has resulted in strong selective pressure for the emergence of antibiotic-resistant bacteria in food animals [2-5]. In turn, animals and animal production environments have become reservoirs for antibiotic-resistant bacteria [6]. Many of these feed additive antibiotics are identical or related to those used in human medicine [7,8]. The largest fraction of medically important antibiotics as feed additives in the USA is used in hogs (69%), compared to 19% in broiler chickens and 12% in beef cattle [9]. Antibiotic resistant bacteria are released into the environment in animal feces and can then spread to other ecological habitats, including humans [6,10,11]. A connection between antibiotic resistance in bacterial isolates from healthy food animals and clinical isolates of human and animal origins has been suggested; however, this is a controversial issue because the ecology of these bacteria and their genes in the agricultural and urban environment is not well understood [10,12-16].

Insects associated with food animals, especially house flies (*Musca domestica*) and German cockroaches (*Blattella germanica*) are not only important nuisance pests but also potential vectors of animal and human pathogens [17,18]. Organic waste in and around animal production facilities provide excellent habitats for the growth and development of these insects. Because of their habitat preferences, unrestricted movement, mode of feeding, and attraction to residential areas, house flies and cockroaches have a great potential to disseminate fecal bacteria, including human and animal pathogens and antibiotic resistant strains [17,18]. With continuing urban expansion in agriculturally zoned areas in the last two decades, there is an increasing concern in the medical and public health community about insect pests directly associated with the spread of bacterial pathogens and antibiotic resistant microorganisms within animal production systems and to residential settings.

Enterococci are ubiquitous Gram-positive, lactic acid bacteria found in various habitats, including the intestinal tract of animals, from insects (10^2 ^to 10^4 ^CFU per house fly) to humans (10^4 ^to 10^6 ^CFU per gram of stool/feces), and environments contaminated by animal or human fecal material as well as in food and feed products derived from animals [19-25]. While some enterococci are used as probiotics, other *Enterococcus *species are important opportunistic and nosocomial pathogens of humans, causing urinary tract infections, bacteremia, intra-abdominal and pelvic infections, wound and tissue infections, and endocarditis [26]. The genus *Enterococcus *presently comprises over 30 species; however, *E. faecalis *and *E. faecium *are the two major species of clinical importance [20]. Enterococci are considered a reservoir of antibiotic resistance genes to a wide range of antibiotics (including beta-lactams and high concentration aminoglycosides) frequently used to treat infections of Gram-positive cocci. Enterococci have been implicated in dissemination of antibiotic resistance and virulence genes both intra- and interspecifically because of their ability to acquire and transfer antibiotic resistance through transfer of plasmids and transposons. In addition, enterococcal acquisition of vancomycin resistance leaves few options for therapeutic management [26-31]. Several studies have highlighted the importance of enterococci as a reservoir of antibiotic resistance genes in the environment [22,26,27,32,33]. However, little information is available about the role of insects in the ecology and dissemination of antibiotic resistant enterococci in the animal production environment and consequently in animal and public health.

The objective of this study was to determine the prevalence of antibiotic resistant and potentially virulent enterococci in house flies and German cockroaches collected from two commercial swine farms and to compare these to enterococci isolated from swine feces. This is the first comprehensive analysis of antibiotic resistance and virulence of enterococci associated with insect pests in swine farms, and it will enhance our understanding of the role of insects in the ecology of antibiotic resistant and virulent bacteria and in the public health and pre-harvest food safety and security.

## Results

### Prevalence, concentration, and diversity of enterococci

**Figure 1:**
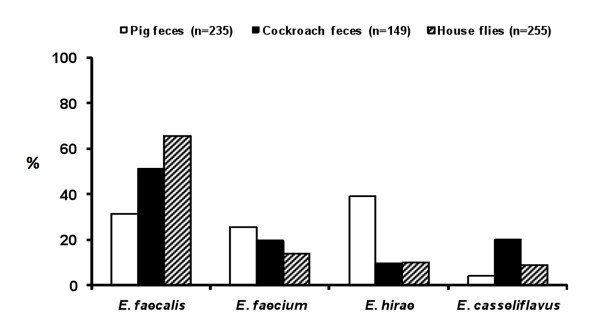
**Diversity of enterococci isolated from pig feces, German cockroach feces, and the digestive tract of house flies collected on two swine farms**. The percent prevalence was calculated for each bacterial species within the three sources.

Enterococci from pig fecal samples (*n *= 119), German cockroaches fecal samples (*n *= 83), and digestive tract of house flies (*n *= 162), collected from two commercial swine farms, were isolated, quantified, identified, and screened for antibiotic resistance and virulence by a polyphasic approach (phenotypic and genotypic analysis). Enterococci were detected in 106 (89.1%) pig fecal samples, 78 (94.0%) cockroach fecal samples, and the digestive tracts of 159 (98.1%) house flies collected from swine farms. The concentration of enterococci (mean ± SEM) was 4.2 ± 0.7 × 10^4 ^CFU/house fly, 5.5 ± 1.1 × 10^6 ^CFU/g of cockroach feces, and 3.2 ± 0.8 × 10^5 ^CFU/g of pig feces. A total of 639 out of 932 (68.6%) enterococcal isolates from all sources (house flies, cockroaches, and pigs) were successfully identified by multiplex or single PCR to species level. The unidentified isolates (31.4%) were not included in the additional analysis in this study. Although differences in species prevalence varied by sources, *E. faecalis *was the common enterococcal species in all samples (55.5%), followed by *E. hirae *(24.9%), *E. faecium *(12.8%), *E. casseliflavus *(6.7%). The largest number of *E. faecalis *and *E. casseliflavus *isolates was detected in flies and cockroach feces and the highest number of *E. faecium *and *E. hirae *was found in pig feces (Figure 1). Concentration of *E. faecalis *from the digestive tract of house flies was significantly higher compared to that from feces of German cockroaches and pigs and *E. hirae *was significantly more prevalent in pig feces than in roach feces and house flies (Figure 1).

### Prevalence and diversity of antibiotic resistance by phenotype and genotype

**Figure 2:**
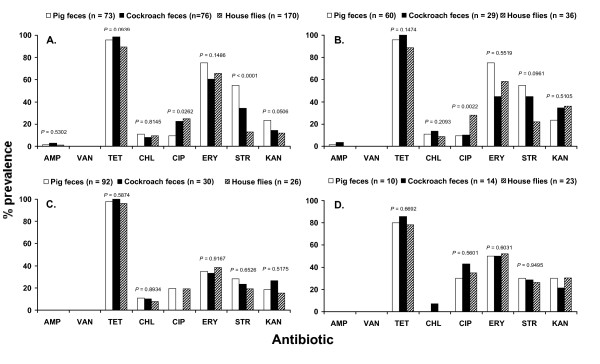
**Phenotypic antibiotic resistance profiles (%) of (A) *E. faecalis*, (B) *E. faecium*, (C) *E. hirae *and (D) *E. casseliflavus *isolated from pig feces, German cockroach feces, and the digestive tract of house flies collected on two swine farms**. AMP = ampicillin, VAN = vancomycin, TET = tetracycline, CHL = chloramphenicol, CIP = ciprofloxacin, ERY = erythromycin, STR = streptomycin, KAN = kanamycin.

The prevalence of antibiotic resistance (expressed as percentages) within each *Enterococcus *spp. isolated from pig and cockroach feces and the digestive tract of house flies is shown in Figure 2. Among the isolates tested, no vancomycin resistance was observed and only a few isolates of *E. faecalis *and *E. faecium *were resistant to ampicillin. The majority of identified isolates from all samples showed high prevalence of tetracycline resistance (Tet^r^) followed by resistance to erythromycin (Ery^r)^) (Figure 2). High-level resistance to the aminoglycosides streptomycin and kanamycin was also detected in *E. faecalis*, *E. faecium*, *E. hirae *and *E. casseliflavus *from all samples (Figure 2). In general, the antibiotic resistance profiles of enterococci isolated from pig feces, cockroach feces, and the digestive tract of house flies were similar and no significant differences were observed within the same bacterial species (Figure 2). However, significant differences in resistance to ciprofloxacin and streptomycin were detected in *E. faecalis *(Figure 2A). Likewise, the incidence of ciprofloxacin resistance in *E. faecium *from the digestive tract of house flies was significantly higher compared to *E. faecium *from feces of German cockroaches and pigs (Figure 2B).

The most common combination or resistance traits was Tet^r ^and Ery^r ^(*E. faecalis*, 65.8%; *E. faecium*, 52.0%; *E. hirae*, 34.5%; *E. casseliflavus*, 51.1%), followed by the combination of Tet^r^, Ery^r^, Str^r^, and Kan^r ^(*E. faecalis*, 6.4%; *E. faecium*, 17.6%; *E. hirae*, 8.8%; *E. casseliflavus*, 17.0%). Further, the prevalence of the most common two-antibiotic-resistant isolates (Tet^r ^and Ery^r^) was not significantly different in the feces of pigs and cockroaches and in the digestive tract of house flies (*P *= 0.0816). Similarly, no significant differences (*P *= 0.0596) in the prevalence of multiple-antibiotic-resistant isolates (Tet^r^, Ery^r^, Str^r^, and Kan^r^) were observed among all samples (pig feces, 11.9%; cockroach feces, 10.7%; house flies, 7.5%).

**Figure 3:**
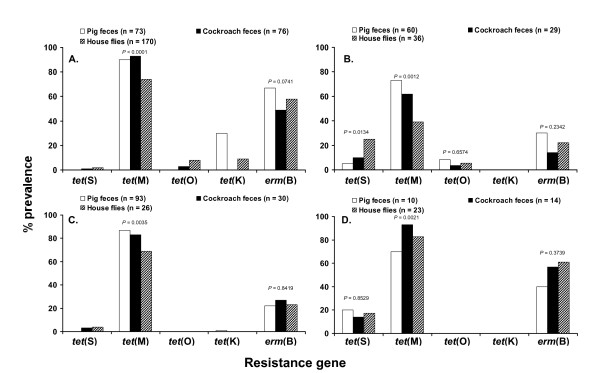
**Genotypic antibiotic resistance profiles (%) of (A) *E. faecalis*, (B) *E. faecium*, (C) *E. hirae *and (D) *E. casseliflavus *isolated from pig feces, German cockroach feces, and the digestive tract of house flies collected on two swine farms**.

The prevalence of resistance genes (expressed as percentages) within each *Enterococcus *spp. is presented in Figure 3. The results revealed that the *tet*(M) and *erm*(B) determinants were widespread, *tet*(S), *tet*(O) and *tet*(K) were rare, and *tet*(A), *tet*(C), *tet*(Q) and *tet*(W) were not detected from the isolates tested based on our PCR approach. Irrespective of their origin, the majority of identified isolates contained the *tet*(M) determinant followed by the *erm*(B) determinant (Figure 3). Significant differences in prevalence of the *tet*(M) determinant were detected in enterococci isolated from pig and cockroach feces and the digestive tract of house flies (Figure 3). In contrast, the *erm*(B) determinant was equally prevalent in enterococci isolated from these three sources and no significant differences were observed within the same bacterial species (Figure 3).

The distribution and combination of resistance genes in phenotypically resistant enterococci are shown in Tables 1, 2, and Additional files 1-3). Many *E. faecalis *(93.4%), *E. faecium *(81.2%), and *E. casseliflavus *(90.9%) carried at least one resistance determinant. Among the isolates tested, the most common determinant was the ribosomal protection protein mechanism encoded by *tet*(M), alone or in combination with other determinants (Tables 1, 2, and Additional files 1-2). No significant differences were found in the prevalence of the *tet*(M) gene alone in *E. faecium *(*P *= 0.2837), *E. hirae *(*P *= 0.0823) and *E. casseliflavus *(*P *= 0.1223) isolated from pig feces, cockroach feces and the digestive tract of house flies (Tables 1, 2, and Additional file 1). The prevalence of *tet*(M) alone in *E. casseliflavus *from pig and cockroach feces was significantly higher (*P *= 0.0012) compared to that from digestive tracts of house flies (Additional file 2).

**Table 1:** Distribution of *tet*(M), *tet*(O), *tet*(S), *tet*(K) and *erm*(B) determinants in *E. faecalis *isolates from pig feces (n = 73), German cockroach feces (*n *= 76) and house fly digestive tracts (*n *= 170)

Combination of determinants	Number (%) of isolates			Correlation with phenotype (%)		
	
	Pig feces	Cockroach feces	House Flies	Pig feces	Cockroach feces	House Flies
*tet*(M) only	21 (28.8)	35 (46.1)	39 (22.9)	90.5	97.4	94.3
*tet*(O) only	-	-	1 (0.6)	-	-	66.6
*tet*(K) only	-	-	8 (4.7)	-	-	100
*tet*(S) only	-	-	1 (0.6)	-	-	100
*erm*(B) only	3 (4.1)	2 (2.6)	11 (6.5)	100	50.0	92.3
*tet*(M) + *erm*(B)	24 (32.9)	33 (43.4)	66 (38.8)	100/87.5	100/90.0	100/98.4
*tet*(O) + *erm*(B)	-	-	3 (1.8)	-	-	100/100
*tet*(S) + *erm*(B)	-	-	1 (0.6)	-	-	100/100
*tet*(K) + *erm*(B)	1 (1.4)	-	-	100/100	-	-
*tet*(M) + *tet*(O)	-	1 (1.3)	3 (1.8)	-	100	100
*tet*(M) + *tet*(O) + *erm*(B)	-	1 (1.3)	7 (4.1)	-	100/100	100/100
*tet*(M) + *tet*(K) + *erm*(B)	21 (28.8)	-	8 (4.7)	100/95.2	-	100/87.5
*tet*(M) + *tet*(S)+ *erm*(B)	-	1 (1.3)	2 (1.2)	-	100/100	100/100
Isolates with no detected *tet *and *erm*(B) determinants	3 (4.1)	3 (3.9)	20 (11.8)	100/100	33.3/66.6	70.0/80.0

**Table 2:** Distribution of *tet*(M), *tet*(O), *tet*(S), *tet*(K) and *erm*(B) determinants in *E. faecium *isolates from pig feces (*n *= 60), German cockroach feces (*n *= 29) and house fly digestive tracts (*n *= 36).

Combination of determinants	Number (%) of isolates			Correlation with phenotype (%)		
	
	Pig feces	Cockroach feces	House Flies	Pig feces	Cockroach feces	House Flies
*tet*(M) only	29 (48.3)	16 (55.2)	13 (36.1)	100	100	87.5
*tet*(O) only	5 (8.3)	0	0	100	-	-
*tet*(S) only	2 (3.3)	2 (6.9)	8 (22.2)	100	100	100
*erm*(B) only	2 (3.3)	0	0	100	-	-
*tet*(M) + *erm*(B)	15 (25.0)	2 (6.9)	5 (13.9)	100/89.7	100/50	80.0/60.0
*tet*(S) + *erm*(B)	1 (1.7)	1 (3.4)	1 (2.8)	100/100	100/100	100/100
*tet*(O) + *erm*(B)	0	1 (3.4)	1 (2.8)		100/100	100/100
*tet*(M) *+ tet*(O) + *tet*(S) + *erm*(B)	1 (1.7)	0	1 (2.8)	100/100	-	100/100

Isolates with no detected *tet *and *erm*(B) determinants	6 (10.0)	7 (24.1)	8 (22.2)	66.6/66.6	0.0/50.0	25.0/37.5

Multiple resistance determinants, specifically *tet*(M) and *erm*(B), were detected in *E. faecalis, E. faecium*, *E. hirae*, and *E. casseliflavus *(Tables 1, 2, Additional files 1-2). In general, the levels of prevalence of multiple resistance determinants *tet*(M) and *erm*(B) were similar and no significant differences were observed in *E. faecalis *(*P *= 0.4151), *E. faecium *(*P *= 0.0864), *E. hirae *(*P *= 0.5873) and *E. casseliflavus *(*P *= 0.5760) isolated from the digestive tract of house flies and feces of German cockroaches and pigs (Tables 1, 2, Additional files 1-2).

Since most of the tetracycline resistant isolates were also resistant to erythromycin, and the *tet*(M) gene is frequently linked with the *erm*(B) gene on the highly mobile conjugative transposon Tn*1545*, tests for the detection of *int *genes were also performed for the presence of conjugative transposons of the Tn*1545*/Tn*916 *family. The results revealed that the Tn*1545*/Tn*916 *conjugative transposon family was found in 219/639 (34.3%) identified isolates from all samples. The Tn*1545*/Tn*916 *family determinant was commonly detected in *E. faecalis *followed by *E. hirae*, *E. casseliflavus*, and *E. faecium *(Additional file 3). The most common *E. faecalis *genotypes based on a combination of antibiotic resistance and Tn*1545*/Tn*916 *family determinants were *tet*(M) plus *erm*(B) plus Tn*916*/*1545 *followed by *tet*(M) plus Tn*916*/*1545 *(Additional file 3). In addition, many (23.3%) *E. faecalis *isolates from pig feces also carried frequently resistance determinants including *tet*(M), *tet*(K) and *erm*(B) in combination with the Tn*1545*/Tn*916 *family (Additional file 3).

### Prevalence and diversity of virulence factors by phenotype and genotype

**Figure 4:**
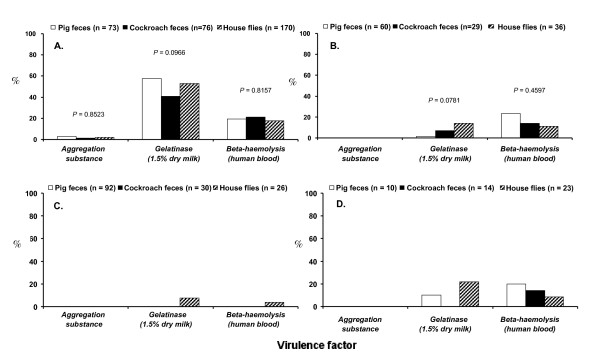
**Phenotypic virulence factor (% prevalence) of (A) *E. faecalis*, (B) *E. faecium*, (C) *E. hirae *and (D) *E. casseliflavus *isolated from pig feces, German cockroach feces, and the digestive tract of house flies collected on two swine farms**.

The overall prevalence of putative virulence factors (gelatinase, haemolysin and aggregation substance production) for all identified isolates is listed in Figure 4. Gelatinase production on skimmed milk agar was the most common virulence factor among all identified isolates, with significantly higher incidence in *E. faecalis *than in *E. casseliflavus*, *E. faecium*, and *E. hirae *(Figure 4). No significant differences were detected in prevalence of gelatinase production among *E. faecalis *and *E. faecium *isolated from the digestive tract of house flies and feces of German cockroaches and pigs (Figure 4).

The prevalence of β-hemolysis on human blood agar in *E. faecalis *was higher than that observed in *E. faecium*, *E. casseliflavus*, and *E. hirae *(Figure 4). In general, the prevalence of β-hemolysis among identified enterococci isolated from pig feces, German cockroach feces and the digestive tract of house flies were similar and no significant differences were observed within the same species (Figure 4).

The clumping/aggregation assay revealed that the prevalence of the clumping phenotype among *E. faecalis *was low as only 6 of the 631 *E. faecalis *(1.95%) isolates aggregated *in vitro*. However, no significant differences were found in the prevalence of this virulence factor among *E. faecalis *isolated from pig feces, German cockroach feces and the digestive tract of house flies (Figure 4A).

**Figure 5:**
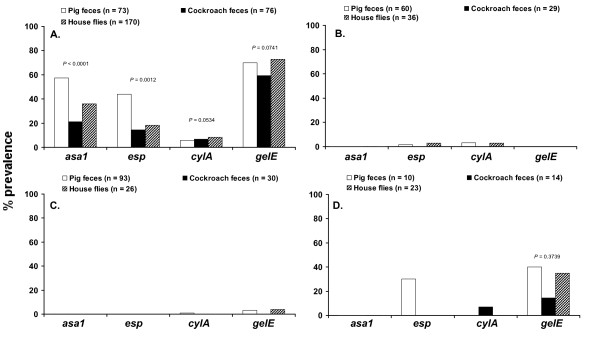
**Distribution of virulence determinants (% prevalence) in (A) *E. faecalis*, (B) *E. faecium*, (C) *E. hirae *and (D) *E. casseliflavus *isolated from pig feces, German cockroach feces, and the digestive tract of house flies collected on two swine farms**.

PCR amplifications of enterococcal DNA with the specific primers for *asa1*, *esp*, *cylA*, and *gelE *revealed significantly higher prevalence of virulence determinants in *E. faecalis *than in other enterococcal species irrespective of the origin of the isolates (Figure 5). *E. faecium *and *E. hirae *isolates were generally without virulence determinants. No significant differences were detected in the prevalence of virulence determinants *gelE *and *cylA *among *E. faecalis *isolated from pig feces, German cockroach feces and the digestive tract of house flies (Figure 5A). However, the prevalence of *asa1 *and *esp *genes in *E. faecalis *from pig feces was significantly higher compared to *E. faecalis *from the digestive tract of house flies and feces of German cockroaches (Figure 5A).

Phenotypic tests showed that the 63.0% of *E. faecalis *that carried *gelE *were gelatinolytic. The test for detection of β-hemolysis in *E. faecalis *revealed there was a 100% (pig feces and cockroach feces) and 92.9% (house flies) correlation between *cylA *and β-hemolysis on human blood. In addition, 8.1% of the *E. faecalis *from house flies was β-hemolytic but negative for *cylA*.

### Genotyping by pulsed-field gel electrophoresis (PFGE)

**Figure 6:**
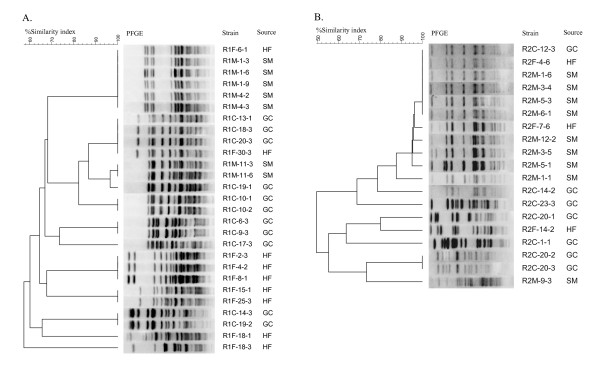
**Dendrograms based on *Apa*I restriction pattern resolved by pulsed-field gel electrophoresis (PFGE) depicting the relationships of A) 27 *E. faecalis *strains and B) 19 *E. faecium *strains isolated from swine manure (SM), house flies (HF), and German cockroaches (GC) from one commercial swine farm**. The scale indicates the level of pattern similarity.

Genotyping of randomly selected *E. faecalis *and *E. faecium *isolated from swine manure, house flies, and German cockroaches from one of the farms revealed that insects and swine manure shared some of the same enterococcal clones. For example, the same genotype of *E. faecalis *was detected from the house fly (strain R1F-6-1) and swine manure (strains R1M-1-3, 1-6, 1-9, 4-2, 4-3) (Figure 6A). Another identical PFGE profile of *E. faecalis *was found in the German cockroach (R1C-13-1, 18-3, 20-3) and in the house fly (R1F-30-3) (Figure 6A). The same clone of *E. faecium *was detected in the German cockroach (R2C-12-3), in the house fly (R2F-4-6), and in swine manure (R2M-1-6, 3-4, 5-3, 6-1) (Figure 6B).

## Discussion

The worldwide increase in the emergence and spread of antibiotic resistance has become a major public health concern, with economic, social and political ramifications. Clearly, the prevalence of antibiotic resistant bacteria in the gastro-intestinal microbial communities of domestic food animals and their feces/manure has become high in the United States likely due to extensive use of antibiotics in food animal production [3,6,10,34-36]. Although a connection between antibiotic resistance in bacterial isolates from healthy food animals and clinical isolates of human and animal origins has been suggested, this is a controversial issue because little is known about the amplification and spread of antibiotic resistant bacteria and genes in the environment [12-14,16,37-41].

The two groups of insects most frequently screened for food borne-pathogens are house flies and cockroaches. These insects have been implicated as mechanical or biological vectors for bacterial pathogens including *Salmonella *spp., *Campylobacter *spp; *Pseudomonas aeruginosa*, *Listeria *spp., *Shigella *spp*., Aeromonas *spp*., Yersinia pseudotuberculosis*, *Escherichia coli *O157:H7, and *E. coli *F18 that can cause diseases in humans and/or animals [17,18]. Multi-antibiotic resistant enterococci have been reported from house flies collected from fast-food restaurants [19]. In addition, the horizontal transfer of *tet(M) *among *E. faecalis *in the house fly digestive tract as well as the great capacity of house flies to contaminate human food with enterococci have been demonstrated [42,43]. Organic wastes in and around animal production facilities including swine farms provide excellent habitats for house flies and German cockroaches. Several features of house flies and cockroaches, including their dependence on live microbial communities, active dispersal ability and human-mediated transport, attraction to places where food is prepared and stored, developmental sites, and mode of feeding/digestion make these insects an important "delivery vehicle" for transport of bacteria including antibiotic resistant enterococci from reservoirs (animal manure), where they pose minimal hazard to people, to places where they pose substantial risk (food) [17,18,44]. Several reports showed a positive correlation between the incidence of food-borne diarrhea and the density of house fly or cockroach populations. For example, suppression of flies in military camps in the Persian Gulf resulted in an 85% decrease in Shigellosis and a 42% reduction in the incidence of other diarrheal disease [45]. Esrey [46] reported a 40% reduction in the incidence of diarrheal infections in children after suppression of a fly population. Another study showed that fly control could reduce trachoma and diarrhoea among children in Gambia [47]. An outbreak of gastro-enteritis caused by *S. typhimurium *in the children's ward of a Belgian hospital dropped as soon as the German cockroach infestation had been controlled [48]. Tarshis [49] recorded that control of cockroaches was accompanied by a decrease in the incidence of endemic infectious hepatitis. The German cockroach was also shown as a potential mechanical vector of the piglet pathogen *Escherichia coli *F18 [50].

To our knowledge, surveillance for resistance to antibiotics in enterococci from insects associated with swine production environments has not been previously conducted. Recently, Graham *et al. *[51] reported that flies may be involved in the transmission of drug resistant enterococci and staphylococci from confined poultry farms. In our study, enterococci were detected in the digestive tracts of house flies, cockroach fecal samples and pig fecal samples collected from two different swine farms with enterococci recovered from 93.7% of 364 samples analyzed. High concentrations of enterococci in the digestive tract of house flies and cockroaches suggest that enterococci are common commensals of these insects intestinal microbiota. Among the four most frequently identified species, *E. faecalis *and *E. faecium *are the most important enterococcal species from a clinical perspective [20,22,27]. However, infections caused by *E. hirae *and *E. casseliflavus *may also occur and warrant attention [52]. In addition, enterococci are regarded as important reservoirs of antibiotic resistance and virulence genes that are often found on mobile genetic elements [22,27,30,52].

The most frequently encountered enterococcal species in the intestines of farm animals are *E. faecalis*, *E. faecium*, *E. hirae, *and *E. durans*; however, culture methods may influence the recovery and selection of enterococcal species [36,53]. The dominance of *E. hirae *in pig feces in our study is consistent with studies of the enterococcal community of swine [32,33]. *E. faecalis *was observed more frequently from the digestive tract of insects and these results are also in agreement with previous studies [19,54]. The favorable conditions in the fly and cockroach digestive tract may serve to select and amplify environmentally acquired *E. faecalis*, including those carrying antibiotic resistance genes.

High frequency of resistance to tetracycline, erythromycin, streptomycin, kanamycin, and ciprofloxacin in our study likely reflects use of tetracyclines, macrolides, aminoglycosides and fluoroquinolones for swine in the USA [55]. Unfortunately, we were unable to obtain any specific information on the use of antibiotics in the two commercial farms in this study. Similar results were reported on antimicrobial resistant phenotypes and resistance genes in enterococci from animals and insects [10,19,51]. The patterns of antibiotic resistance observed in *Enterococcus *spp. recovered from the pig fecal samples were similar to those observed in isolates recovered from digestive tracts of house flies and cockroach fecal samples indicating that insects acquired enterococci from the pig manure. PFGE analysis of selected *E. faecalis *and *E. faecium *isolates confirmed that both insect species carried some of the same clones that were detected in the swine manure. This supports our data indicating that insects acquired the drug-resistant and potentially virulent enterococci from the swine feces although the opposite route cannot be ruled out. However, our previous study [56] showed that the prevalence of antibiotic resistant enterococci in house flies decreases with increasing distance from the likely source (cattle feedlot). This indicates that the source of antibiotic resistant enterococci in house flies and cockroaches in this study was the swine manure due to very high prevalence of antibiotic resistant enterococci in all three sources. The absence of VRE in this study is in agreement with previous findings and reflects a relationship between extensive use of specific antibiotics as growth promoters and presence of VRE [32,35,57]. Since avoparcin has not been used as a growth promoter in the United States, and VRE are rarely isolated from US food animal production environments. In contrast, VRE have been frequently isolated from food animal production environments in Europe where vancomycin was extensively used for farm animals [58].

Our findings are in agreement with the results of other studies which showed that *tet*(M) and *erm*(B) are the most widespread resistance genes among enterococci from food animals or foods [10,15,19,24,59,60]. Furthermore, a strong association of the *tet*(M) and *erm*(B) genes with the conjugative transposon family Tn*1545*/Tn*916 *was also detected in many isolates in our study, indicating that antibiotic resistant enterococci associated with the confined swine environment could be a reservoir of transferable tetracycline and erythromycin resistance. The similar prevalence of resistance determinants and Tn*1545*/Tn*916 *transposons among isolates from pig feces, house flies and cockroach feces indicates exchange of resistant strains or their resistance genes. This is important because the Tn*1545*/Tn*916 *family has a very broad host range and members of this family of transposons can be transferred by conjugation to numerous bacterial species in the human gastrointestinal microbial community [61-63].

The highest incidence of multiple virulence factors was detected in *E. faecalis *with similar virulence profiles from the digestive tract of house flies, cockroach feces and pig feces. The *gelE *gene was detected frequently in *E. faecalis *(63.0%) and was the most common of the virulence factors. Prevalence of the *gelE *gene has been frequently documented in *E. faecalis*, and rarely in *E. faecium *and *E. durans *[12,27]. The presence of *gelE *was, however, not strictly correlated with the phenotype suggesting that some *gelE *genes are silent which is likely due to a 23.9-kb chromosomal deletion involving the *fsr *locus that regulates *gelE *expression [64,65]. We found little correlation between the clumping phenotype *in vitro *and the presence of the *asa1 *gene in *E. faecalis *showing that *asa1 *is not commonly expressed under these *in vitro *conditions. The phenotypic test for β-hemolysis (cytolysin production) with *E. faecalis*, *E. faecium *and *E. casseliflavus *showed a strong correlation between *cylA *and β-hemolysis on human blood. However, 8.1% of the *E. faecalis *from house flies were positive for β-hemolysis but negative for *cylA*, suggesting the presence of unknown determinant(s). Some of the genes encoding virulence determinants, including cytolysin and aggregation substance, are known to be present on pheromone-responsive plasmids, such as pAD1 and therefore transferable to other *E. faecalis *strains [27].

The data presented in this study offer evidence that should be helpful for future research initiatives aimed at reducing the dissemination of antibiotic resistant and virulent bacteria. It is likely that the high prevalence of resistant and potentially virulent enterococci in house flies and German cockroaches associated with confined swine environments reflects an extensive use of antibiotics by the swine industry. However, the degree to which these resistant and virulent enterococci hamper the efficacy of medically important antibiotics and thus pose risks to humans is unknown. The gastrointestinal tracts of mini-pigs, humans, and mice provide favorable environments for intra- and interspecies transfer of antibiotic resistance genes, but these processes have not been investigated in the digestive tract of insects and related arthropods with few exceptions [42,66-71]. Knowing the sources of enterococci harboring in house flies and German cockroaches is also important to accurately assess risk, to identify and implement management plans for fecal waste, and to establish insect management practices that prevent the spread of antibiotic resistant strains and other potential human and animal pathogens. Further studies are warranted to pinpoint the potential sources of fecal contamination of insects, their subsequent contamination of food and feed, and for a detailed understanding gene transfer in the digestive tract of insects.

## Conclusion

In summary, our study showed that multi-antibiotic resistant and potentially virulent enterococci are prevalent in confined swine production (in pig feces, house flies and German cockroaches). House flies and German cockroaches likely serve as vectors and/or reservoirs for antibiotic resistance and virulence genes in the confined swine production environment and consequently they present animal and public health risks. Therefore, effective management strategies aimed at reducing insect pest populations should be an important component of pre-harvest food safety efforts in the future, with increasing recognition of enterococci as human opportunistic pathogens.

## Methods

### Sample collection and isolation of enterococci

All samples were collected from two commercial farms, one in Duplin county, North Carolina and one in Ottawa county, Kansas. House flies (*Musca domestica*) were collected using a sweep net. Individual house flies were surface sterilized with sodium hypochlorite and ethanol [44], homogenized in 1 ml of phosphate buffered saline (PBS), serially diluted, and drop-plated onto modified Enterococcus agar (mENT, Becton Dickinson, MA, USA). German cockroaches (*Blattella germanica*) were collected by brushing them into sterile plastic bags. Cockroaches were randomly divided among sterile plastic petri dishes (20 per petri dish) and allowed to produce feces overnight at room temperature. Fecal material (10 mg) from each petri dish was aseptically collected and processed as below. Pig feces were aseptically collected in sterile 50 ml Falcon tubes. One gram of feces was suspended in 9 ml of PBS and vortexed. An aliquot of 1 ml from each suspension was serially diluted in PBS and drop-plated onto mENT agar. All inoculated mENT agar plates were incubated at 37°C for 48 h. Purple/red bacterial colonies with a morphology characteristic of enterococci were counted, and up to four presumptive enterococcal colonies per sample were sub-cultured on trypticase soy agar (TSA; Becton Dickinson, MA, USA) incubated at 37°C for 24 h. Presumptive enterococcal colonies were identified at the genus level with the esculin hydrolysis test using Enterococcossel broth (Becton Dickinson, MA, USA) incubated for 24 h at 44°C [72]. Isolates confirmed as enterococci were streaked on TSA and incubated for 24 h at 37°C and stored at 4°C for further analysis.

### Enterococcal species identification

Species-level identification was performed using multiplex PCR for four common species: *E. faecalis, E. faecium, E. casseliflavus *and *E. gallinarum *and single PCR for *E. hirae *[73-75]. Control strains consisting of *E. faecalis *ATCC 19433, *E. faecium *ATCC 19434, *E. gallinarum *ATCC 49579, *E. *c*asseliflavus *ATCC 25788, and *E. hirae *ATCC 8043 were included with each PCR assay. *E. mundtii *ATCC 43186 was used as negative control.

### Phenotypic screening for antibiotic resistance and virulence factors

All identified isolates were tested for sensitivity to six antibiotics using standard disc diffusion method. Antibiotic discs of ampicillin (AMP, 15 μg/ml), vancomycin (VAN 30 μg/ml), tetracycline (TET, 30 μg/ml), chloramphenicol (CHL, 30 μg/ml), ciprofloxacin (CIP, 5 μg/ml), and erythromycin (ERY, 15 μg/ml) (all Oxoid) were used. High levels resistance to streptomycin (STR) and kanamycin (KAN) were assessed by the agar dilution technique using 2,000 μg/ml of streptomycin or kanamycin in brain heart infusion agar (Becton Dickinson, MA, USA). The protocols followed the guidelines of the National Committee for Clinical Laboratory Standards [76]. *E. faecalis *ATCC 19433, *E. faecium *ATCC 19434, *E. gallinarum *ATCC 49579 and *E. casseliflavus *ATCC 25788 were used as quality control strains.

Gelatinase activity was detected by streaking all identified isolates on TSA containing 1.5% (v/v) skim milk [27]. *E. faecalis *MMH594 was used as a positive control and *E. faecalis *FA2-2 as a negative control.

For detection of hemolytic activity, *E. faecalis *and *E. faecium *were streaked on Columbia agar base supplemented with 5% (v/v) fresh sterile human blood and grown for 24-48 h at 37°C. Isolates showing a complete clearance zone around the colonies indicated β-hemolysin production [27]. *E. faecalis *MMH594 was used as a positive control and *E. faecalis *FA2-2 as a negative control.

Production of aggregation substance was determined by the clumping assay [77]. *E. faecalis *OG1RF:pCF10 and JH2-2 were used as positive and negative controls, respectively.

### Genotypic screening for antibiotic resistance, virulence and integrase genes

Multiplex or single PCR were used to screen all identified isolates for tetracycline and erythromycin resistance genes including, *tet*(S), *tet*(M), *tet*(O), *tet*(K), *tet*(A), *tet*(C), *tet*(Q), *tet*(W)] and *erm*(B) and for four putative virulence determinants *gelE *, *cylA, esp*, and *asa1 *[78-81]. Integrase gene (*int*) was used for detection of the conjugative transposon family Tn*1545*/Tn*916 *[19,82]. To confirm the identity of our PCR products, one randomly selected PCR product for each resistance, virulence, and transposon determinant was purified with GFX PCR DNA and Gel Band Purification Kit (Amersham Bioscience, UK) and sequences were determined on an ABI 3700 DNA Analyzer at the K-State DNA Sequencing Facility using the same PCR primers. Sequences were analyzed for similarity to known sequences in the GenBank database using BLAST (Basic Local Alignment Search Tool) [83]. Manual sequence alignment was done with CodonCode Aligner (Version 1,3,4) (CodonCode Corporation, Dedham, MA) (data not shown).

### Genotyping of selected isolates with pulsed-field gel electrophoresis (PFGE)

PFGE protocol of Amachawadi *et al*. [84] was used with minor modifications. Agarose plugs were digested with 40 U of *Apa*I (Promega, Madison, WI) for 4 h at 37°C. The digested plugs were run on to a 1% SeaKem Gold Agarose (Lonza, Rockland, MI) gel using CHEF Mapper (Bio-Rad, Hercules, CA) with initial pulse time for 1 s and final time for 20 s at 200 V for 21 h. Cluster analysis was performed with BioNumerics software (Applied Maths, Korrijk, Belgium) using the band-based Dice correlation coefficient and the unweighted pair group mathematical average algorithm (UPGMA).

### Data analysis

Differences in the prevalence of antibiotic resistance and virulence factors (genotype and phenotype) among enterococcal isolates from pig feces, house flies and roach feces were analyzed using chi-square analysis of contingency tables and Fisher's exact test (*α = *0.05). Species with zero prevalence of antibiotic resistance and virulence factors (genotype and phenotype) were not included in the analysis.

## Authors' contributions

LZ and CS designed the study. AA and AG performed the analysis. AA, CS, AG, and LZ wrote the manuscript. All authors approved the final manuscript.

## Supplementary Material

Additional file 1 **Distribution of *tet*(M), *tet*(S), *tet*(K) and *erm*(B) determinants in *E. hirae *isolates from pig feces (*n *= 93), German cockroach feces (*n *= 30) and house fly digestive tracts (*n *= 26)**. Table describing distribution of *tet *and *erm *genes in *E. hirae *from various sources and their correlation with the phenotype. Click here for file

Additional file 2 **Distribution of *tet*(M), *tet*(S) and *erm*(B) determinants in *E. casseliflavus *isolates from pig feces (*n *= 10), German cockroach feces (*n *= 14) and house fly digestive tracts (*n *=23)**. Table describing distribution of *tet *and *erm *genes in *E. casseliflavus *from various sources and their correlation with the phenotype. Click here for file

Additional file 3 **Distribution [number (%) of isolates] of the tetracycline resistance genes, *erm*(B) gene, and Tn*916*/*1545 *family among isolates from pig feces, cockroach feces and the digestive tract of house flies**. Table describing combinations of antibiotic resistance determinants and transposon Tn*916*/*1545 *family in four *Enterococcus *species isolated from various sources. Click here for file
