# Supplementary material for: Insects in confined swine operations carry a large antibiotic resistant and potentially virulent enterococcal community
Source: BMC Microbiol. 2011 Jan 26;11:23. doi: 10.1186/1471-2180-11-23 (PMC3039560; doi:10.1186/1471-2180-11-23)
Supplement: Additional file 1 — Distribution of tet(M), tet(S), tet(K) and erm(B) determinants in E. hirae isolates from pig feces (n = 93), German cockroach feces (n = 30) and house fly digestive tracts (n = 26). Table describing distribution of tet and erm genes in E. hirae from various sources and their correlation with the phenotype. [file 1471-2180-11-23-S1.DOCX]

| **Combination of determinants** | **Number (%) of isolates** | | |  | **Correlation with phenotype (%)** | | |
| --- | --- | --- | --- | --- | --- | --- | --- |
|  | **Pig feces** | **Cockroach feces** | **House Flies** |  | **Pig feces** | **Cockroach feces** | **House Flies** |
| *tet*(M) only | 68 (73.1) | 17 (56.7) | 14 (53.8) |  | 100 | 100 | 100 |
| *tet*(K) only | 1 (1.1) | 0 | 0 |  | 100 | - | - |
| *tet*(S) only | 0 | 1 (3.3) | 1 (3.8) |  | - | 100 | 100 |
| *erm*(B) only | 1 (1.1) | 0 | 2 (7.7) |  | 100 | - | 100 |
| *tet*(M) + *erm*(B) | 19 (20.4) | 8 (26.7) | 4 (15.4) |  | 94.7/63.2 | 100/87.5 | 100/100 |
| Isolates with no detected *tet* and *erm*(B) determinants | 4 (4.3) | 4 (13.3) | 5 (19.2) |  | 25.0/75.0 | 0.0/100.0 | 20.0/60.0 |
