# Supplementary material for: Insects in confined swine operations carry a large antibiotic resistant and potentially virulent enterococcal community
Source: BMC Microbiol. 2011 Jan 26;11:23. doi: 10.1186/1471-2180-11-23 (PMC3039560; doi:10.1186/1471-2180-11-23)
Supplement: Additional file 2 — Distribution of tet(M), tet(S) and erm(B) determinants in E. casseliflavus isolates from pig feces (n = 10), German cockroach feces (n = 14) and house fly digestive tracts (n =23). Table describing distribution of tet and erm genes in E. casseliflavus from various sources and their correlation with the phenotype. [file 1471-2180-11-23-S2.DOCX]

| **Combination of determinants** | **Number (%) of isolates** | | |  | **Correlation with phenotype (%)** | | |
| --- | --- | --- | --- | --- | --- | --- | --- |
|  | **Pig feces** | **Cockroach feces** | **House Flies** |  | **Pig feces** | **Cockroach feces** | **House Flies** |
| *tet*(M) only | 3 (30.0) | 6 (42.8) | 4 (17.3) |  | 100 | 100 | 100 |
| *tet*(S) only | 1 (10.0) | 0 | 0 |  | 100 | - | - |
| *erm*(B) only | 0 | 1 (7.1) | 0 |  | - | 100 | - |
| *tet*(M) + *tet*(S) | 0 | 0 | 1 (4.3) |  | - | - | 100 |
| *tet*(M) + *erm*(B) | 3 (30.0) | 5 (35.7) | 11 (47.8) |  | 94.7/63.2 | 100/87.5 | 100/100 |
| *tet*(M) + *tet*(S) + *erm*(B) | 1 (10.0) | 2 (14.3) | 3 (13.0) |  | 94.7/63.2 | 100/87.5 | 100/100 |
| Isolates with no detected *tet* and *erm*(B) determinants | 2 (20.0) | 0 | 4 (17.4) |  | 50.0/50.0 | - | 100/100 |
