# Supplementary material for: Insects in confined swine operations carry a large antibiotic resistant and potentially virulent enterococcal community
Source: BMC Microbiol. 2011 Jan 26;11:23. doi: 10.1186/1471-2180-11-23 (PMC3039560; doi:10.1186/1471-2180-11-23)
Supplement: Additional file 3 — Distribution [number (%) of isolates] of the tetracycline resistance genes, erm(B) gene, and Tn916/1545 family among isolates from pig feces, cockroach feces and the digestive tract of house flies. Table describing combinations of antibiotic resistance determinants and transposon Tn916/1545 family in four Enterococcus species isolated from various sources. [file 1471-2180-11-23-S3.DOCX]

| **Combination of determinants** | ***E. faecalis*** | | | ***E. faecium*** | | | ***E. hirae*** | | | ***E. casseliflavus*** | | |
| --- | --- | --- | --- | --- | --- | --- | --- | --- | --- | --- | --- | --- |
|  | **Pig feces**  **(*n*=73)** | **Cockroach**  **feces**  **(*n*=76)** | **House Flies**  **(*n*=170)** | **Pig feces**  **(*n*=60)** | **Cockroach feces**  **(*n*=29)** | **House Flies**  **(*n*=36)** | **Pig feces**  **(*n*=93)** | **Cockroach**  **feces**  **(*n*=30)** | **House Flies**  **(*n*=26)** | **Pig feces**  **(*n*=10)** | **Cockroach**  **feces**  **(*n*=14)** | **House Flies**  **(*n*=23)** |
| Tn*916/1545* only   | - | 1  (1.3) | 3  (1.8) | - | 1  (3.4) | - | 1  (1.1) | - | 4  (17.4) | 1  (10.0) | - | 3  (13.0) |
| *tet*(M) | 9  (12.3) | 15 (19.7) | 12  (7.1) | 2  (3.3) | 1  (3.4) | 1  (2.8) | 23 (24.7) | 3  (10.0) | - | 1  (10.0) | 2  (14.3) | 2  (8.7) |
| *tet*(O) | - | - | 1  (0.6) | - | - | - | - | - | 1  (4.3) | - | - | - |
| *tet*(K) | - | - | 2  (1.2) | - | - | - | - | - | - | - | - | - |
| *erm*(B) | 1 (1.4) | 1  (1.3) | 11  (6.8) | - | - | - | - | - | - | - | - | - |
| *tet*(M) + *erm*(B) | 12 (16.4) | 16 (21.1) | 26 (15.3) | 4  (6.6) | - | 3  (8.3) | 9  (9.7) | 1  (3.3) | 3  (13.0) | - | 4  (28.6) | 3  (13.0) |
| *tet*(O)+ *erm*(B) | - | - | 1  (0.6) | - | - | - | - | - | - | - | - | - |
| *tet*(K)+ *erm*(B) | 1  (1.4) | - | - | - | 1  (3.4) | - | - | - | - | - | - | - |
| *tet*(M)+ *tet*(O) + *erm*(B) | - | - | 1  (0.6) | - | - | - | - | - | - | - | - | - |
| *tet*(M)+ *tet*(S) + *erm*(B) | - | 1  (1.3) | 1  (0.6) | - | - | - | - | - | - | - | - | 3  (17.4) |
| *tet*(M)+ *tet*(K) + *erm*(B) | 17 (23.3) | - | 7  (4.1) | - | - | - | - | - | - | - | - | - |
| At least one determinant  but no Tn*916/1545* | 30  (41.1) | 40  (52.6) | 88  (51.8) | 48  (80.0) | 20  (68.9) | 24  (66.7) | 57  (60.2) | 22  (73.3) | 17  (65.4) | 7  (70.0) | 8  (57.1) | 11  (47.8) |
| Isolates with no detected determinants | 3  (4.1) | 2  (2.6) | 17  (10.0) | 6  (10.0) | 6  (20.9) | 8  (22.2) | 3  (3.2) | 4  (13.3) | 1  (3.8) | 1  (10.0) | 0  (0.0) | 1  (52.2) |
